# Evidence of Extensive Alternative Splicing in Post Mortem Human Brain HTT Transcription by mRNA Sequencing

**DOI:** 10.1371/journal.pone.0141298

**Published:** 2015-10-23

**Authors:** Adam T. Labadorf, Richard H. Myers

**Affiliations:** 1 Department of Neurology, Boston University School of Medicine, Boston, Massachusetts, United States of America; 2 Bioinformatics Program, Boston University, Boston, Massachusetts, United States of America; 3 Genome Science Institute, Boston University School of Medicine, Boston, Massachusetts, United States of America; University of California, Los Angeles, UNITED STATES

## Abstract

Despite 20 years since its discovery, the gene responsible for Huntington’s Disease, *HTT*, has still not had its function or transcriptional profile completely characterized. In response to a recent report by Ruzo et al. of several novel splice forms of *HTT* in human embryonic stem cell lines, we have analyzed a set of mRNA sequencing datasets from post mortem human brain from Huntington’s disease, Parkinson’s disease, and neurologically normal control subjects to evaluate support for previously observed and to identify novel splice patterns. A custom analysis pipeline produced supporting evidence for some of the results reported by two previous studies of alternative isoforms as well as identifying previously unreported splice patterns. All of the alternative splice patterns were of relatively low abundance compared to the canonical splice form.

## Introduction

Huntington’s Disease (HD) is caused by a mutant huntingtin protein (htt) protein that contains a polyglutamine tract encoded by a trinucleotide CAG repeat in the first exon of the *HTT* gene. Despite twenty years of study following the discovery of *HTT* [1], the transcriptional species of the gene have not been fully characterized in human patients and the function of its encoded protein is incompletely understood. Alternative splicing (AS) occurs when different combinations of exons are spliced together before translation to protein. *HTT* is known to undergo AS in both humans and model organisms [2–5], but the extent of alternative splicing is not established. Recently, Ruzo et al [2015] report five new splice isoforms of the *HTT* gene using neuronally differentiated human embryonic stem cell lines. To investigate whether evidence for these isoforms is found in post mortem human brain, we analyzed a set of mRNA-Seq samples generated from prefrontal cortex (BA9) of 28 Huntington’s disease (HD), 29 Parkinson’s disease (PD), and 50 neuropathologically normal control brains (C) for alternative splicing. We constructed an *HTT* “superset” by concatenating all of the reads for all samples together in order to best characterize the constitutive splicing events across all conditions independent of disease, as well as concatenating samples within conditions to look for disease-specific effects. Using a custom analysis pipeline (see Methods) we observed previously reported splice forms and identified putative novel alternative splicing events in the spliced read patterns of the sequencing data. Nearly all alternative splice forms were very lowly abundant compared to the primary transcript pattern but confidently detected by multiple reads.

## Results

### Intronic reads are abundant and variable

**Fig 1:**
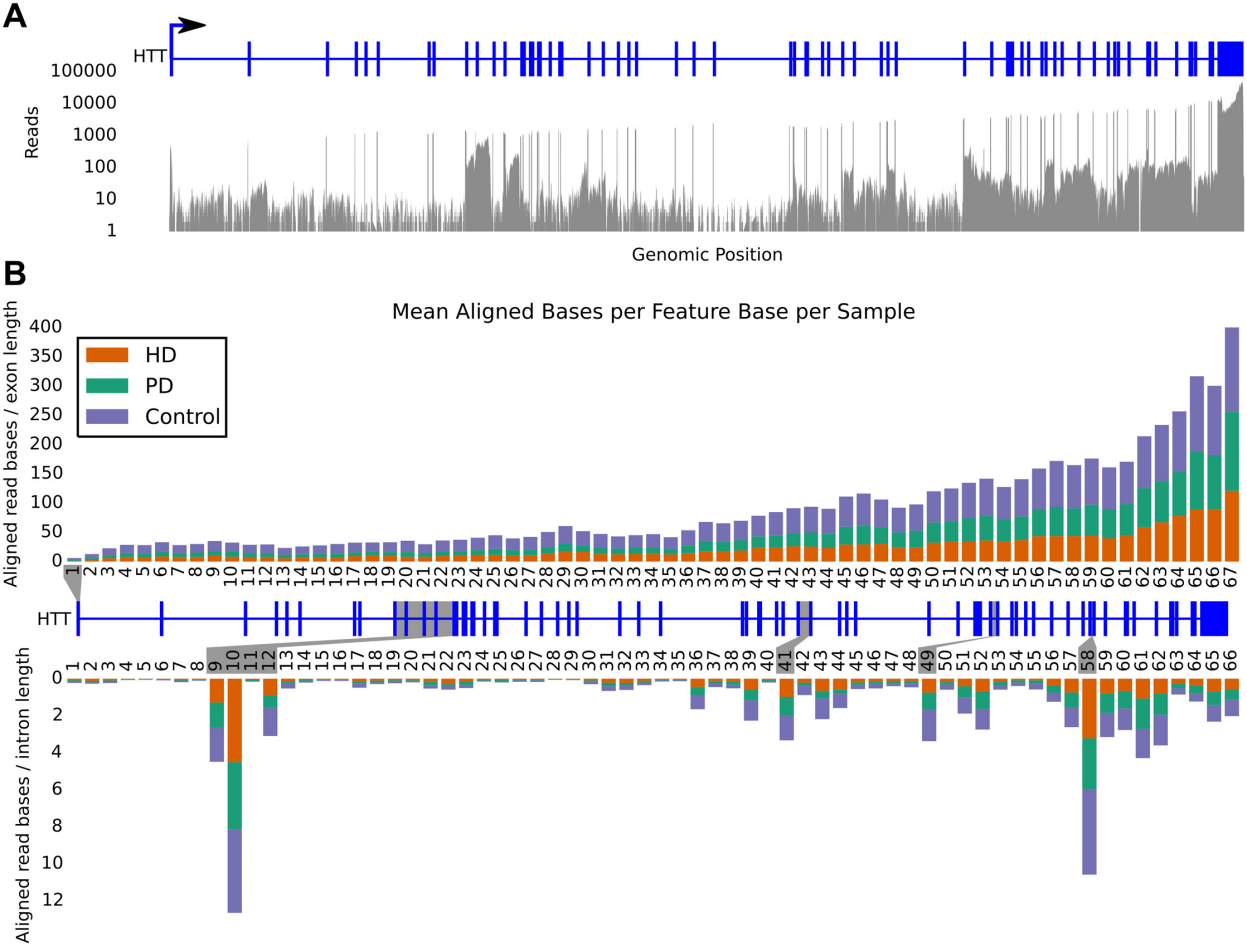
A) Read pileup of the superset reads showing coverage in exons and specific introns. Canonical HTT gene model is in blue. B) Relative contribution of reads from each disease dataset to the superset binned into exon (top) and intron (bottom) features. Each bar is the average number of covered bases per base position in the given feature divided by the number of samples in the corresponding condition. The canonical gene model lies between the bar charts. High intronic coverage is apparent in introns 9, 10, 12, 41, 49, and 58, highlighted in grey. None of the features are obviously biased toward any of the conditions. The rightward skew of counts is indicative of the poly-A selection method used in library prep.

The read pileup of the superset reveals extensive intronic transcription in specific regions of HTT, as shown in Fig 1A. To better quantify the transcriptional abundance and compare among conditions, the canonical splice form of *HTT* (HTT-001) was used to define the splicing pattern and the number of aligned bases in each of the concatenated sets was counted separately for each exon and intron (hereafter called “features”). The number of aligned bases per feature was then divided by the length of the feature to arrive at the average read coverage within each condition, and then averaged over the number of samples in each condition, resulting in the average coverage per feature per sample. Fig 1B depicts this average read coverage for exons and introns. As seen in both subfigures, the level of intronic transcription is highly variable across the body of the gene. In particular, introns 9 and 10 show highly abundant transcripts nearly equal in depth to that of flanking exons. In contrast, other introns including 11 and 34–35 show very little evidence of transcription, while nearly every intron downstream of intron 56 shows some consistent read depth. We examined three other genes to better understand the prevalence of intronic read coverage and found similar trends (Supplement Material section S2).

### Alternative isoforms appear throughout HTT at low relative abundance

**Fig 2:**
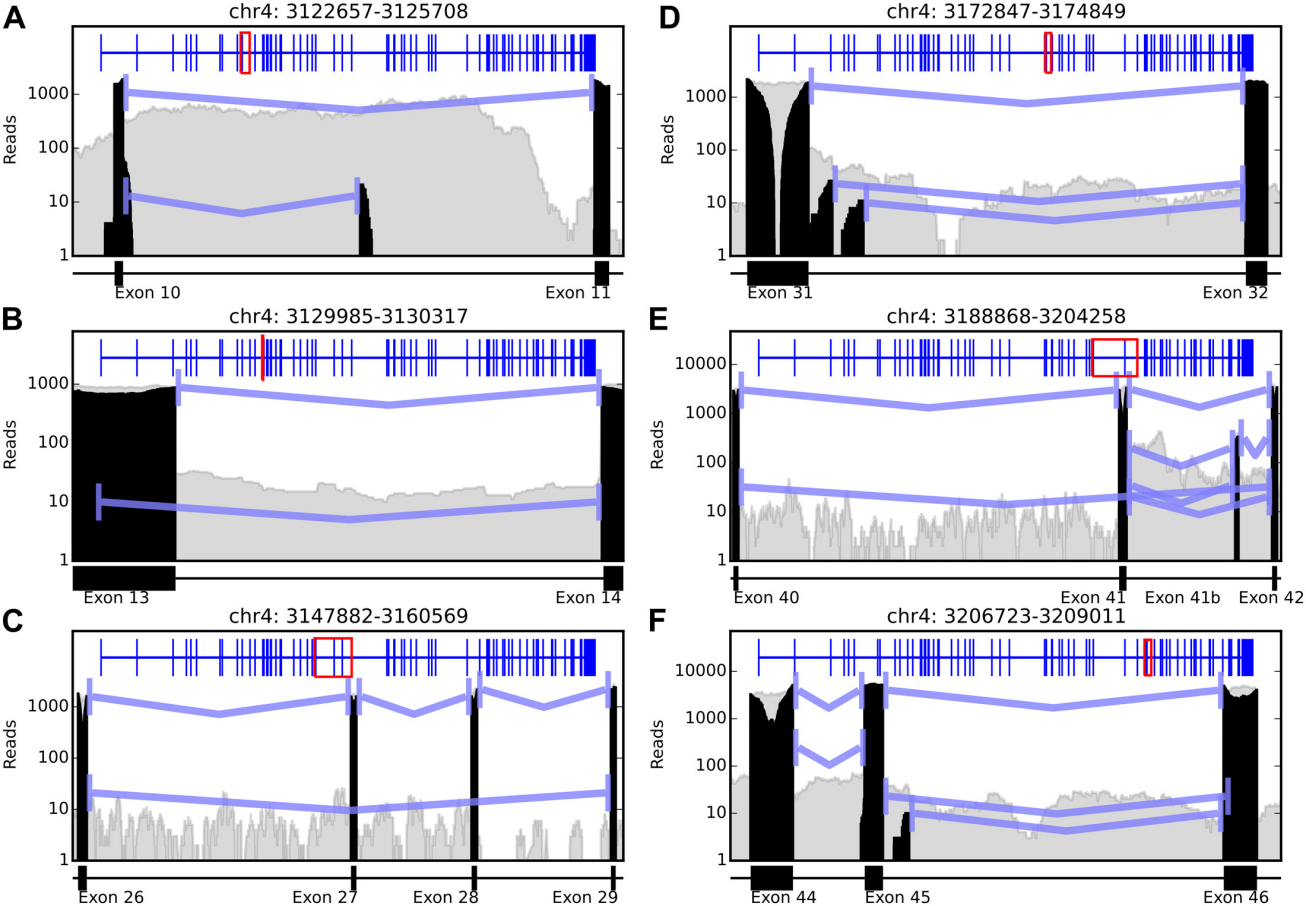
Splicing events detected in reads of both novel and previously reported splice forms. Grey areas indicate overall aligned read coverage in the region, black areas are the spliced reads that contribute to the splicing events. Blue lines indicate detected splicing events with a minimum of 10 supporting reads. The blue track across the top of all plots is the canonical HTT splice form, with red boxes indicating the position in the gene region displayed. Splicing patterns shown in B, E, and F support the spliceforms HTT-d13, HTT-41b, and HTT-d46 reported in Ruzo et al. In C, the skipped exon 28 is consistent with an isoform identified in mouse and human [Hughes JMB 2014] but, in these data, is only seen in a splice pattern where exon 27 is also skipped.

Analysis of the spliced read pattern reveals extensive evidence of alternative splicing events in the HTT gene using the read superset. 85 splicing events total and 25 AS events involving 11 loci were discovered throughout the gene with a minimum of 10 splice read support for each AS event. The AS events suggest that the major alternative splicing types detectable with short sequencing reads exist, including alternative acceptor and donor splice sites, and exon skipping events, and that most alternative splice patterns exist in low-abundance transcripts (intron retention events cannot generally be detected with sequencing data due to long introns and short reads). A selection of splicing events are described in Table 1 and depicted in Fig 2, with additional figures in the supplement (S1 Text Figs A-K). The splice patterns shown in Fig 2 include both novel and previously observed isoforms of *HTT* from both human and mouse models. The patterns in Fig 2B, 2E and 2F support the splice forms HTT-d13, HTT-41b, and HTT-d46 reported in Ruzo et al. In C, the skipped exon 28 is consistent with an isoform identified in mouse and human [3] but is only seen in a splice pattern where exon 27 is also skipped. There is evidence for transcription in introns 9 and 10 in GENCODE v21 [6,7] transcripts HTT-006 and HTT-013, respectively, but the splice junction spanning exon 9 to the middle of intron 9 has not been previously observed. The splice pattern in D is supported partially by the transcript HTT-011 but is annotated as a 3’ UTR in GENOCODE v21 where the splice junction reads suggest this alternative splicing event is an alternate donor site for a longer transcript. The exon skipping event from exon 40 to 42 in E appears to be novel. There is no prior evidence of an alternative donor splice site from exon 45 to 46 as observed in F.

**Table 1:** Detected Alternative Splice Events.

Locus	Fig 2 Label	Splice coordinates	AS Type	Read Support	Previous Evidence
chr4:3,122,657–3,125,708	A	3,122,936–3,124,249	Acceptor	13	
		3,122,936–3,125,548	Canonical	1075	
chr4:3,129,985–3,130,317	B	3,129,999–3,130,304	Donor	10	Ruzo et al
		3,130,047–3,130,304	Canonical	874	
chr4:3,47,882–3,160,569	C	3,148,207–3,160,281	Skipped Exon	21	Hughes et al
		3,148,207–3,154,292	Canonical	1574	
chr4:3,172,847–3,174,849	D	3,173,131–3,174,720	Canonical	1619	
		3,173,218–3,174,720	Donor	23	
		3,173,331–3,174,720	Donor	10	
chr4:3,188,868–3,204,258	E	3,189,093–3,199,731	Canonical	2998	
		3,189,093–3,204,006	Skipped Exon	32	
		3,199,939–3,202,976	Added Exon	194	Ruzo et al
		3,199,939–3,202,981	Added Exon	34	
		3,199,939–3,204,006	Canonical	3069	
		3,199,939–3,204,009	Acceptor	20	
chr4:3,206,723,3,209,011	F	3,206,983–3,207,280	Canonical	3962	
		3,206,983–3,207,285	Acceptor	246	
		3,207,357–3,208,772	Canonical	3995	
		3,207,357–3,208,802	Acceptor	23	Ruzo et al
		3,207,466–3,208,772	Donor	10	

Twelve of the 25 alternative splicing (AS) events detected using the superset reads, as depicted in Fig 2. All AS forms are much less abundant than the canonical splice form. Read Support column lists the number of junction reads supporting the splice junction, with the percentage of total junction reads involved in this event listed in parentheses. The remaining events are included as processed data file GSE71191_all_merged_HTT.bed.gz in the GEO accession GSE71191.

## Discussion

The goal of this study was to seek and evaluate evidence for previously reported alternative *HTT* splicing events in our unique mRNA-Seq datasets from human control and patient post-mortem brains. We found evidence for some but not all of the splice forms reported in Ruzo et al. and Hughes et al., though the low relative abundance of the splice patterns in this dataset does agree with the low transcript abundance found in those studies. Without wet lab identification of isoforms exhibiting the AS events reported here, it is impossible to speculate on the potential coding consequences of these alterations to the canonical splice pattern of *HTT*, but such low abundance of the AS events may suggest they play a limited role compared to the full length protein in the HD or wild type context. In addition to the splice patterns, we also observed consistent intronic transcription in specific introns throughout the entire gene. While it is possible that some of the reads observed originate from unspliced pre-mRNA molecules, the non-random pattern of intronic coverage suggests that these events exist in mature mRNA transcripts. Even though there are 10 introns in the canonical splice form that are shorter than the average fragment length of the paired end reads (~300nt), due to the length of the reads (101nt paired end), it is difficult to verify the existence of retained introns with this data since reads spanning introns could originate from pre-mRNA. We therefore did not attempt to estimate the frequency of intron retention events.

It is difficult to confidently assess differential splice pattern usage between conditions from these data. The poly-A selection technique used to isolate mRNA molecules for sequencing results in 3’ bias of transcript sequencing coverage and very few reads mapping to the 5’ region of *HTT* for individual samples. The low coverage across the features of most of the gene makes statistical differentiation between feature usage challenging. It is also unclear whether current read count normalization techniques, which are necessary to adjust read counts for library size differences to make them comparable, are appropriate for the normalization of the counts in this context. For all of these reasons, we did not seek to identify differential splice pattern usage between conditions. We suggest that wet lab experiments would be a more reliable method to identify these transcripts, but this work is outside the scope of the current study.

Our data support *HTT* as having potentially many alternative splice forms, in agreement with the observation of high levels of AS in humans in general [8], and further investigation into isoforms that may contain the AS events detected here may shed light into the function of this gene and whether these AS events contribute to the pathogenesis of HD.

## Methods

mRNA-Seq from whole-tissue homogenate of post mortem human brain samples from HD, PD, and C individuals were prepared and sequenced as previously described [9]. 101nt paired end sequencing reads (performed on an Illumina’s HiSeq 2000 system at Tufts University sequencing core facility, http://tucf-genomics.tufts.edu/) were first quality-trimmed using the sickle software package [10], aligned to the hg38 human reference[11] with STAR[12], where multimapped reads were assigned unique locations with ORMAN[13]. Only reads aligning primarily to the HTT region were retained for analysis. BAM files containing HTT-aligned reads were merged within each condition and across all conditions using samtools. Prior to initiating our study we reviewed the available methods and found that none of those that we could identify were able to adequately perform this analysis (see S1 Text). We therefore designed and implemented our own method as follows. For counting aligned bases, aligned reads were mapped to *HTT* introns and exons using the GENCODE v21 [6,7] transcript HTT-001 annotation and every aligned base was counted and binned on the feature level using a custom python script. For detecting splicing events, a custom python script was used to analyze all HTT reads. A splicing event is defined as a contiguous span of gap greater than 9 bases in a read alignment along with the specific start and stop locations of the gap. All splicing events with a support of at least 10 reads in the superset were reported. Figures were generated using custom python scripts in ipython notebook [14]. The data used in this paper is publicly available in GEO under accession GSE71191. The code used to perform the analysis and generate all plots is available at https://github.com/adamlabadorf/HTT_AS.

This study has been designated exempt (Protocol # H-28974) by the Boston University School of Medicine Institutional Review Board, as no human subjects were studied and all data are derived from post-mortem human brain specimens.

## Supporting Information

S1 Text Evaluation of existing alternative splicing event detection algorithms and intronic read coverage. Plots of additional previously reported and novel splicing event loci (**Figs A-K**).
(PDF)
