# Supplementary material for: Evidence of Extensive Alternative Splicing in Post Mortem Human Brain HTT Transcription by mRNA Sequencing
Source: PLoS One. 2015 Oct 23;10(10):e0141298. doi: 10.1371/journal.pone.0141298 (PMC4619731; doi:10.1371/journal.pone.0141298)
Supplement: S1 Text — Plots of additional previously reported and novel splicing event loci (Figs A-K). (PDF) [file pone.0141298.s001.pdf]

Evaluation of existing alternative splicing event detection algorithms. Prior to initiating our study we reviewed the available methods and in the end found that all of those that we could identify were not able to address the unique aspects of this study. Our strategy was aimed specifically at maximizing the number of reads available for the purpose of identifying previously reported and novel (previously unrecognized) splicing events of the HTT gene in a condition-agnostic manner. Our goal was to identify novel splicing events, while the purpose of the majority of existing programs (Cuffdiff2[1], DEXSeq[2], MISO[3], MATS[4], SUPPA[5]) is to characterize the difference in isoform usage between conditions. Although the question of whether alternative splicing events are different between conditions is of interest, the low abundance and high complexity of the splice patterns observed in these data makes it difficult to quantify differences between our three conditions with individual samples using currently available tools. Choosing an appropriate normalization methods for concatenated samples is also not straightforward, as discussed below.

To properly quantify differential alternative splicing, library adjustments (e.g. count normalization) must be performed on a per-sample basis to make samples across conditions comparable. By concatenating many or all samples together, the ability to appropriately correct for these sample specific differences is lost. However, as evidenced by the very low abundance of alternatively spliced reads from previously reported splice patterns, we would not have sufficient power to detect many of these splicing events without performing concatenation. Existing tools such as MISO[3] attempt to quantify the relative abundance of isoforms, but because, in the absence of methods to normalize our libraries, these tools would not optimally detect novel splice variants. Attempts at quantifying relative abundance of isoforms are best left to future experiments that specifically target the transcripts identified here (such as transcript targeted qPCR), that more closely and empirically examine the existence and relative abundances of specific transcripts suggested by these data.

Finally, 'state of the art' computational strategies for identifying alternative splicing events use known isoform annotations to detect differential exon boundaries. Most of the programs (and all of the ones we evaluated: Cuffdiff2[1], MISO[3], AltEventFinder[6], MATS[4], SUPPA[5]) require a pre-existing set of isoforms to be supplied for quantification, and suggest that these isoforms be estimated from the data using programs such as cufflinks[7] or Scripture[8] if they are unavailable. We attempted to assemble the HTT transcriptome from these data but, due to the extremely high depth attained through concatenation, the transcript assembly was not accurate. As noted in our manuscript, intronic regions sometimes showed abundant coverage across the body of the gene, which would not be typical of an individual sample, and thus it seems the transcriptome assembly algorithm was confused and yielded likely false exon prediction results. The IGV screenshot below shows a track where the program Scripture identified "exons". There are many false positives, and therefore we did not opt to use exiting transcriptome assembly approaches on these data.

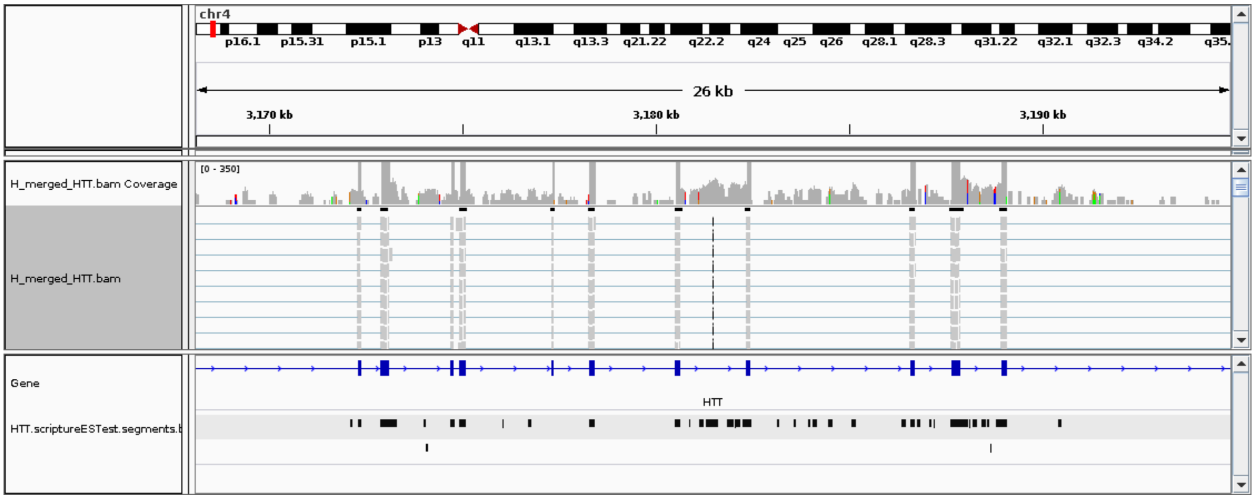

IGV screenshot with results of best Scripture transcriptome assembly of the superset data for a region of HTT. The black track has the regions where Scripture inferred a transcript, clearly demonstrating noise in intronic regions where true exonic expression is unlikely. This is representative of the whole transcriptome assembly for HTT using this data.

Intronic read coverage. We investigated three other genes to better understand the prevalence of intronic read coverage as observed in HTT. We chose three (somewhat arbitrary) genes that had comparable read depth to HTT in the original sequencing experiment (~3000-5000 average reads per sample mapping to the gene locus):

- PON2 (GRCh38 coords ch7:95404067-95436723), which has little alternative isoform evidence
- TPM3 (chr1:154155154-154184567), which has moderate alternative isoform evidence
- CPFS3L (chr1:1311347-1324859), which has extensive alternative isoform evidence

All plots show the read depth for the body of the gene on a log scale. All genes show a similar pattern to HTT, with consistent intronic read coverage that is usually many orders of magnitude lower than flanking exons, with decreasing coverage toward the 5' end of the gene, consistent with a poly-A tail selection. Interestingly, in PON2, where there is not extensive evidence of alternative splicing, we see varying levels of intronic coverage between introns, but within introns the coverage is consistent. The reasons for this are unclear, as the lack of evidence for intron retention events suggests these introns are not translated into protein fragments, but there seems to be no pattern in intronic depth with intron length. There is evidence that the exons flanking the more highly abundant introns can extend into longer transcripts, which may indicate alternative 3' isoforms, but the data here is inconclusive to support this hypothesis.

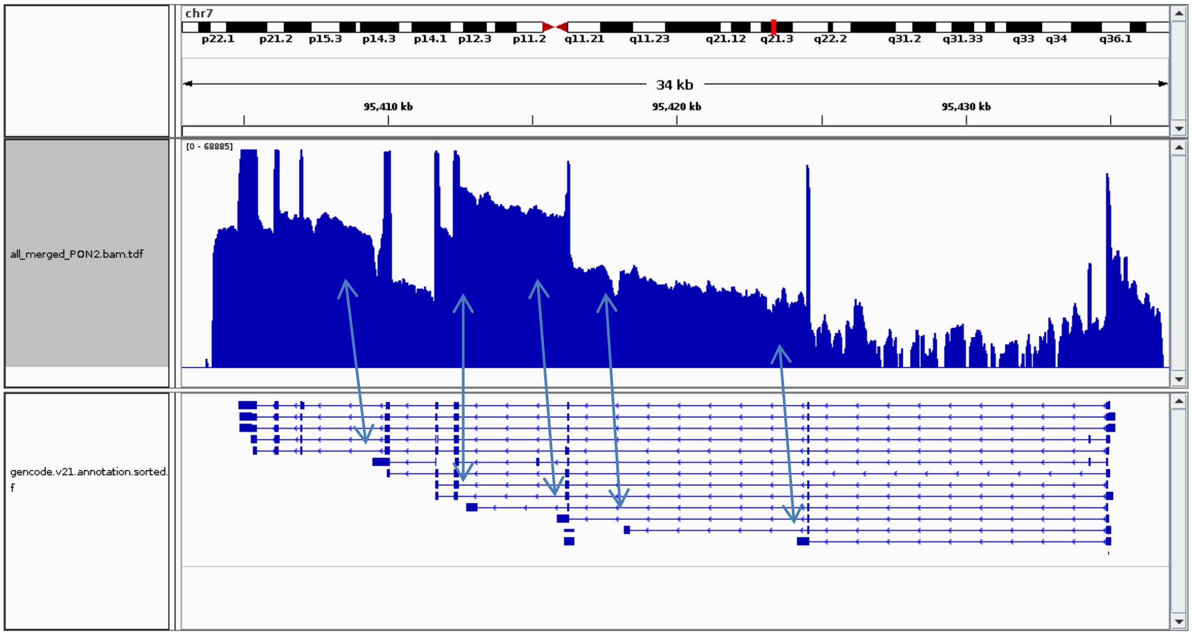

PON2 read coverage from all concatenated datasets. Read pileup in blue has a maximum of 68,885 read depth and is plotted on a log scale . Arrows indicate where annotated transcripts and intronic coverage suggest putative 3' transcripts.

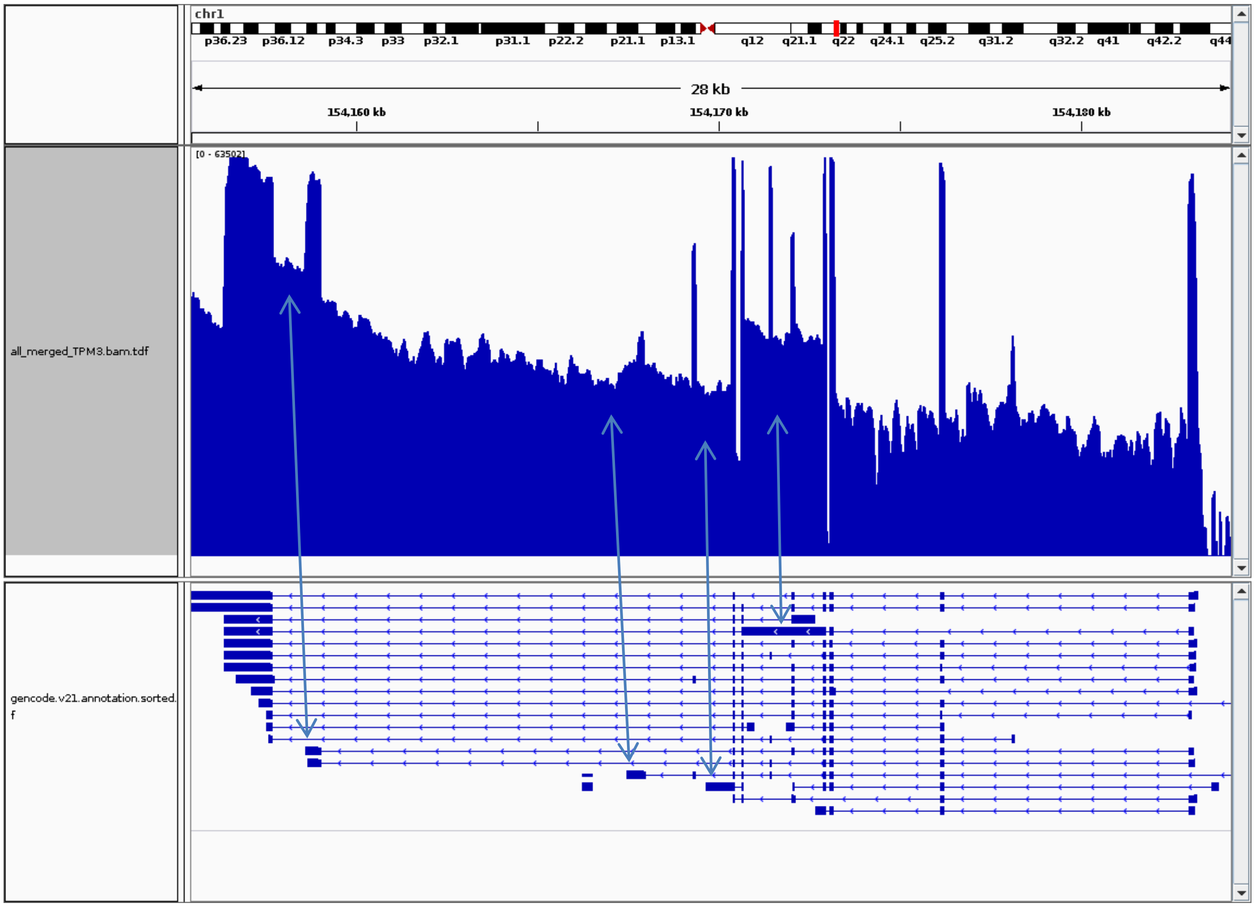

TPM3 read coverage from all concatenated datasets. Read pileup in blue has a maximum of 63,502 read depth and is plotted on a log scale. Arrows indicate intronic evidence of annotated intron retention and alternative 3' UTR events.

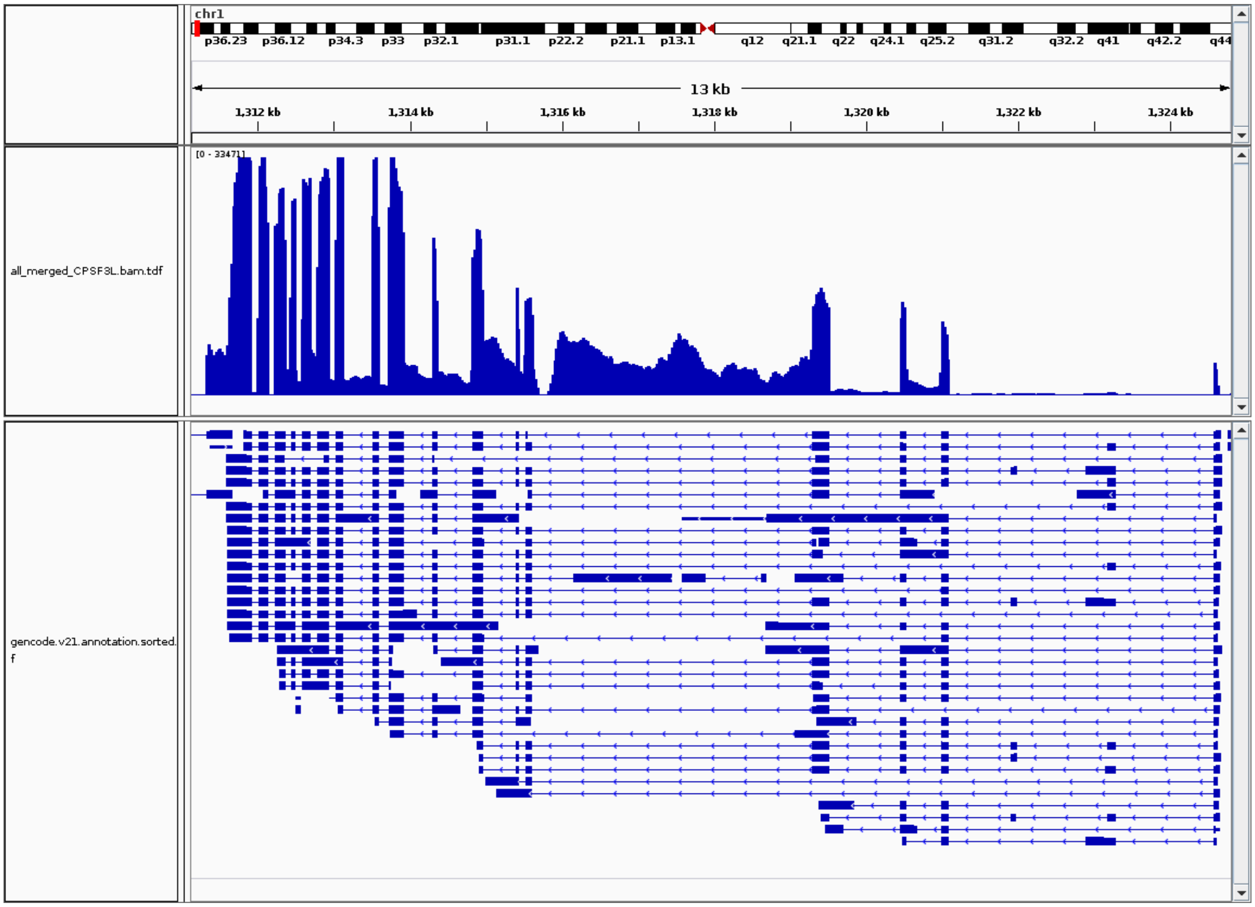

CPSF3L read coverage from all concatenated datasets. Read pileup in blue has a maximum of 33,471 read depth and is plotted on a log scale.

Supplemental Figures A-K. All alternative splicing patterns found in *HTT* that have at least 10 supporting reads. The first subfigure (chr4:3127546-3129987) is a locus reported by Ruzo et al to be alternatively spliced but this pattern is not seen in these data.

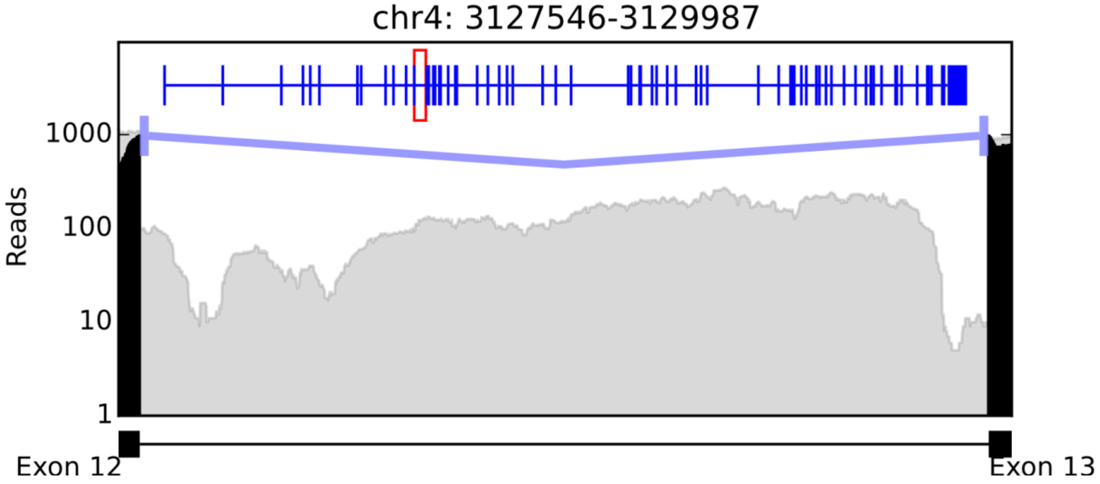

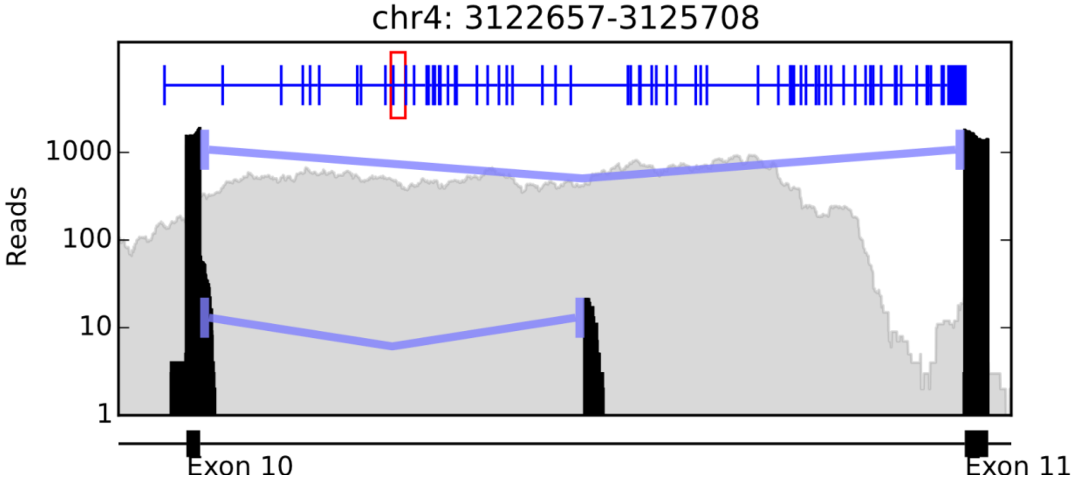

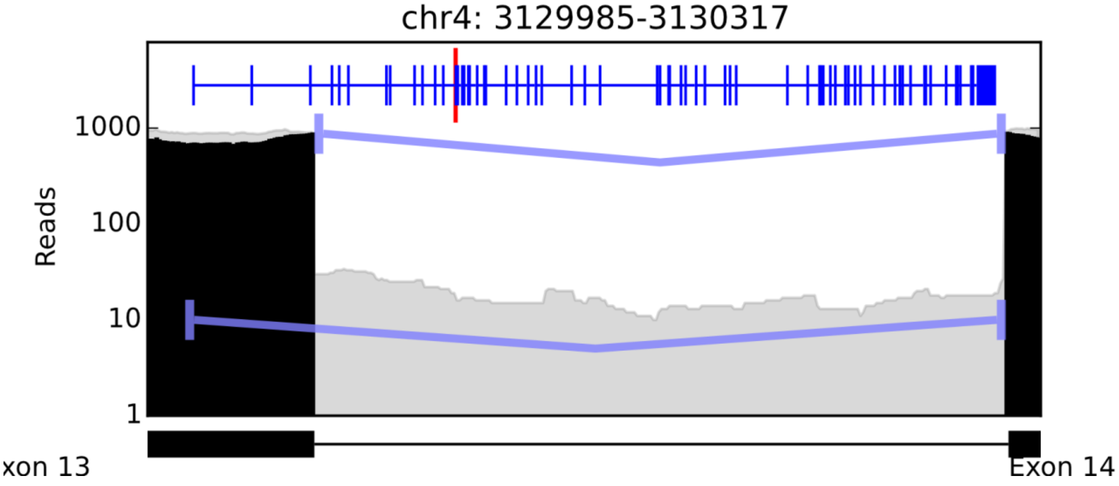

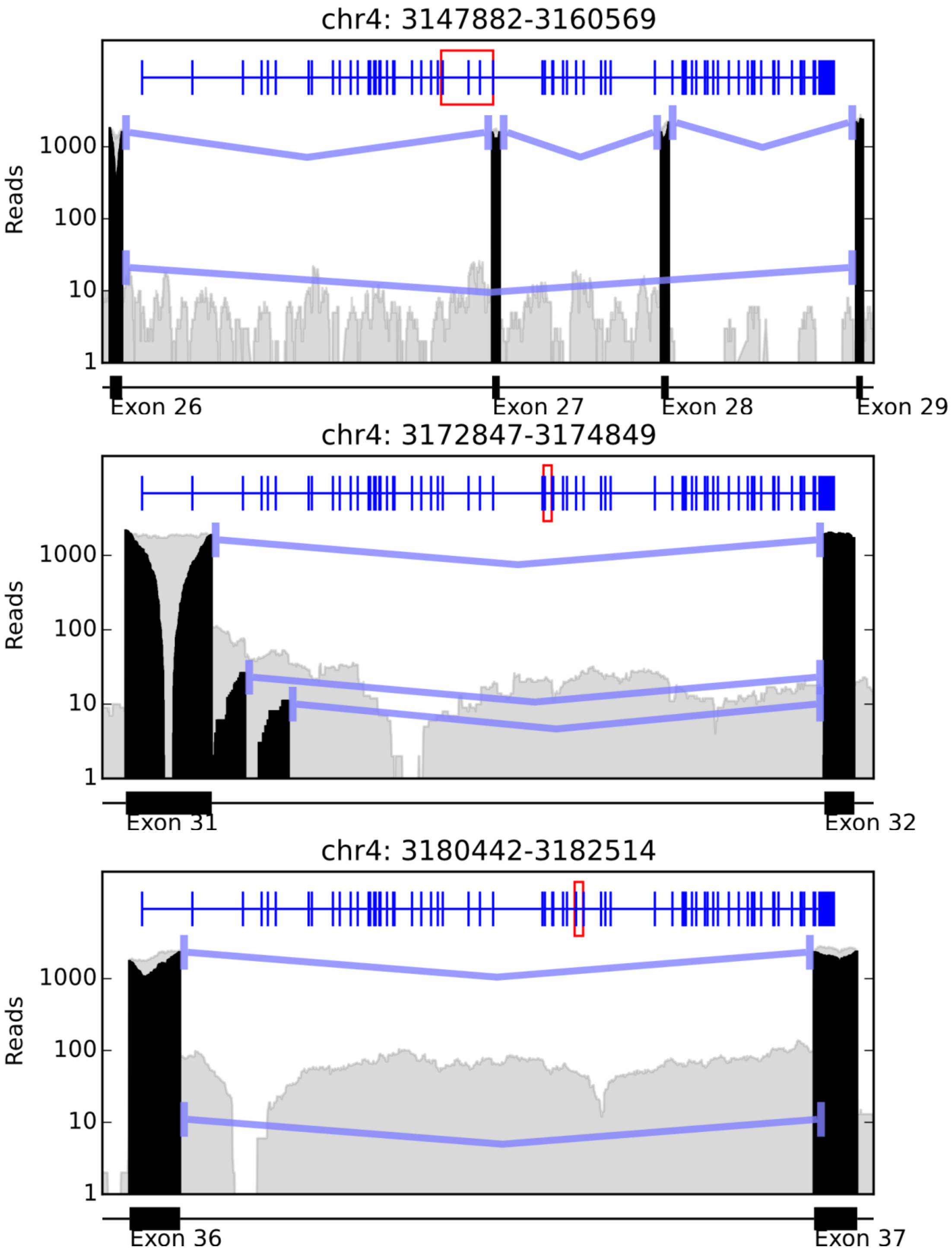
chr4: 3147882-3160569
Reads
1000
100
10
1
Exon 26
Exon 27
Exon 28
Exon 29
chr4: 3172847-3174849
Reads
1000
100
10
1
Exon 31
Exon 32
chr4: 3180442-3182514
Reads
1000
100
10
1
Exon 36
Exon 37

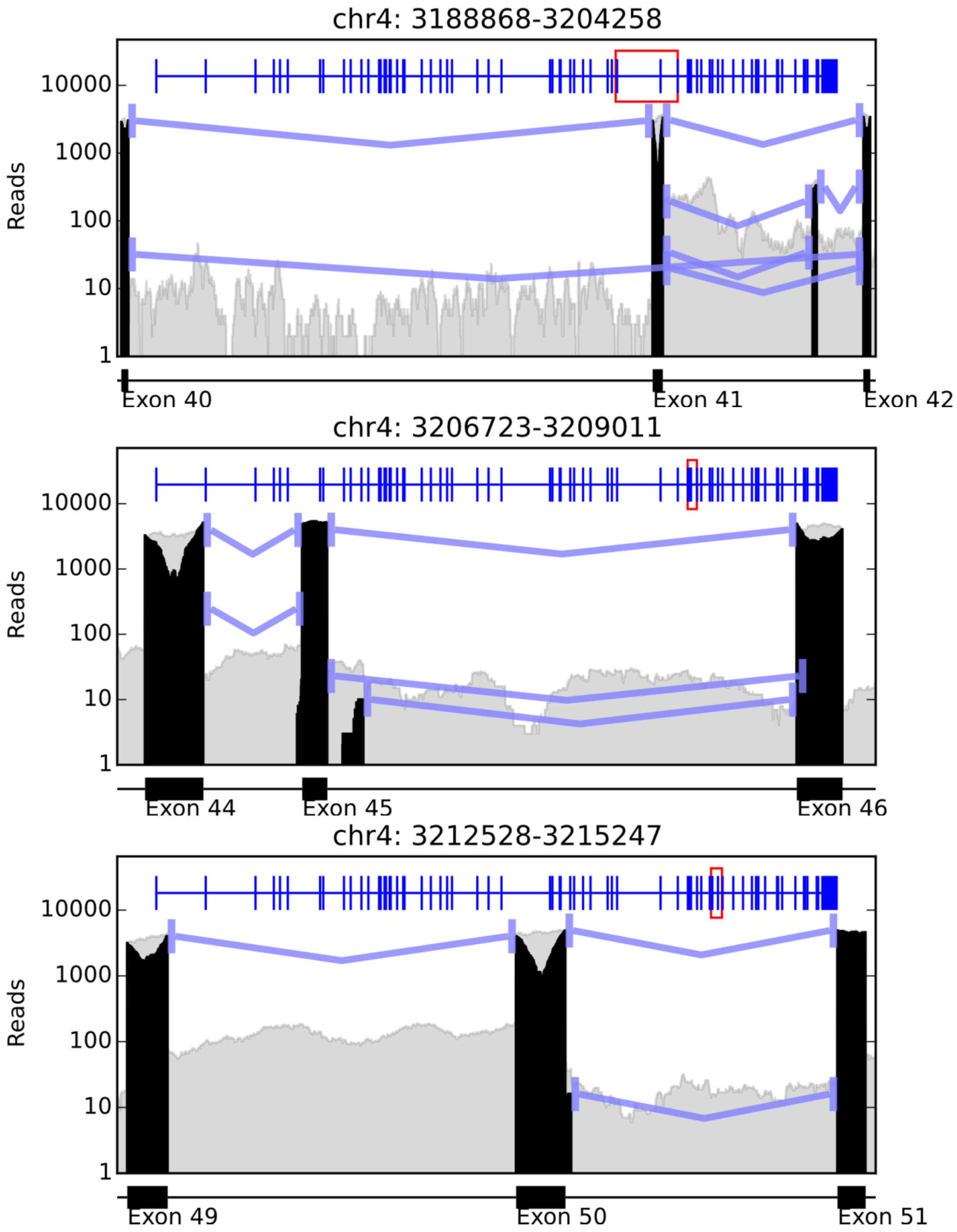
chr4: 3188868-3204258
Reads
10000
1000
100
10
1
Exon 40
Exon 41
Exon 42
chr4: 3206723-3209011
Exon 44
Exon 45
Exon 46
chr4: 3212528-3215247
Exon 49
Exon 50
Exon 51

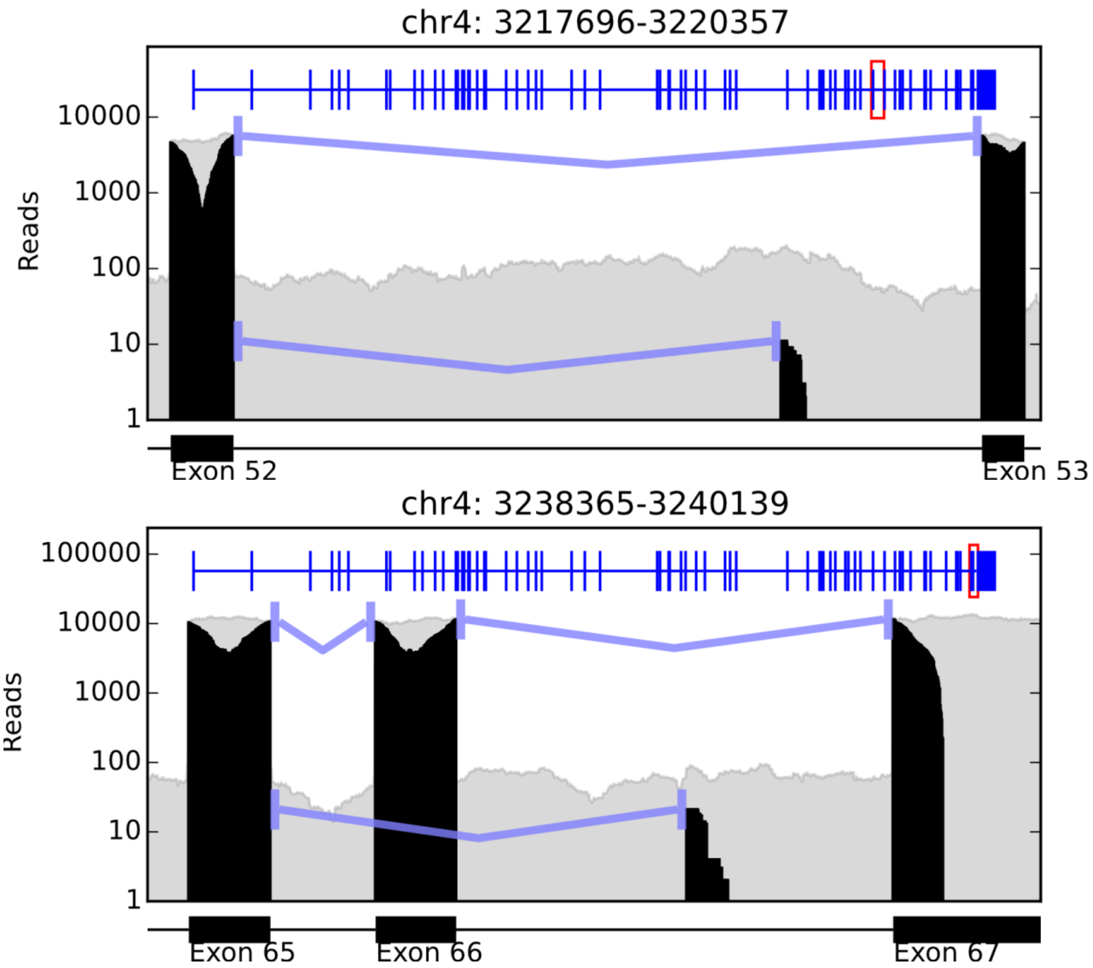
chr4: 3217696-3220357
Reads
10000
1000
100
10
1
Exon 52
Exon 53
chr4: 3238365-3240139
Reads
100000
10000
1000
100
10
1
Exon 65
Exon 66
Exon 67

1. Trapnell C, Hendrickson DG, Sauvageau M, Goff L, Rinn JL, Pachter L. Differential analysis of gene regulation at transcript resolution with RNA-seq. Nat Biotechnol. 2013;31: 46–53. doi:10.1038/nbt.2450

2. Anders S, Reyes A, Huber W. Detecting differential usage of exons from RNA-seq data. Genome Res. 2012;22: 2008–2017. doi:10.1101/gr.133744.111

3. Katz Y, Wang ET, Airoldi EM, Burge CB. Analysis and design of RNA sequencing experiments for identifying isoform regulation. Nat Methods. 2010;7: 1009–1015. doi:10.1038/nmeth.1528

4. Shen S, Park JW, Lu Z, Lin L, Henry MD, Wu YN, et al. rMATS: Robust and flexible detection of differential alternative splicing from replicate RNA-Seq data. Proc Natl Acad Sci. 2014;111: E5593–E5601. doi:10.1073/pnas.1419161111

5. Alamancos GP, Pagès A, Trincado JL, Bellora N, Eyras E. SUPPA: a super-fast pipeline for alternative splicing analysis from RNA-Seq. bioRxiv. 2014; 008763. doi:10.1101/008763

6. Zhou A, Breese MR, Hao Y, Edenberg HJ, Li L, Skaar TC, et al. Alt Event Finder: a tool for extracting alternative splicing events from RNA-seq data. BMC Genomics. 2012;13: S10. doi:10.1186/1471-2164-13-S8-S10

7. Trapnell C, Williams BA, Pertea G, Mortazavi A, Kwan G, van Baren MJ, et al. Transcript assembly and quantification by RNA-Seq reveals unannotated transcripts and isoform switching during cell differentiation. Nat Biotechnol. 2010;28: 511–515. doi:10.1038/nbt.1621

8. Guttman M, Garber M, Levin JZ, Donaghey J, Robinson J, Adiconis X, et al. Ab initio reconstruction of cell type-specific transcriptomes in mouse reveals the conserved multi-exonic structure of lincRNAs. Nat Biotechnol. 2010;28: 503–510. doi:10.1038/nbt.1633
